# Interactive locomotion: Investigation and modeling of physically-paired humans while walking

**DOI:** 10.1371/journal.pone.0179989

**Published:** 2017-09-06

**Authors:** Jessica Lanini, Alexis Duburcq, Hamed Razavi, Camille G. Le Goff, Auke Jan Ijspeert

**Affiliations:** Ecole Polytechnique Fédérale de Lausanne, Lausanne CH-1015, Switzerland; Fondazione Santa Lucia Istituto di Ricovero e Cura a Carattere Scientifico, ITALY

## Abstract

In spite of extensive studies on human walking, less research has been conducted on human walking gait adaptation during interaction with another human. In this paper, we study a particular case of interactive locomotion where two humans carry a rigid object together. Experimental data from two persons walking together, one in front of the other, while carrying a stretcher-like object is presented, and the adaptation of their walking gaits and coordination of the foot-fall patterns are analyzed. It is observed that in more than 70% of the experiments the subjects synchronize their walking gaits; it is shown that these walking gaits can be associated to quadrupedal gaits. Moreover, in order to understand the extent by which the passive dynamics can explain this synchronization behaviour, a simple 2D model, made of two-coupled spring-loaded inverted pendulums, is developed, and a comparison between the experiments and simulations with this model is presented, showing that with this simple model we are able to reproduce some aspects of human walking behaviour when paired with another human.

## 1 Introduction

Extensive studies have investigated the effect of sensory feedback on human walking gait. Zivotofsky et al. [1, 2] have studied different types of sensory feedback, including tactile (e.g., holding hands) and have shown that different types of sensory feedback, such as visual, acoustic, and tactile cause synchronous walking; the tactile feedback was found to be the most effective in causing synchronization. Also, in [3] the interpersonal synchronization between pairs of subjects walking side-by-side (without holding hands) on a treadmill was shown to have spells of attraction to certain phase relations, but no strict phase locking was found, implying that the interlimb coupling without haptic feedback is weaker.

Moreover, biomechanical properties of partners, such as leg length, influence the strength of synchronization during unintentional gait entrainment. Nessler et al. [4] found that among all the pairs walking side-by-side on two treadmills under different sensory feedback conditions, those ones who exhibited unilateral step frequency locking (a.k.a. entrainment) had significantly lower leg length differences between the partners. Moreover, a significant correlation was found between leg length difference and both difference in stepping frequency and frequency locking.

Even though, as presented in some of the work above, side-by-side walking has been well analyzed by considering different sensory feedbacks and anthropometric parameters, to the best of our knowledge the effect of the haptic interaction through an object between two people, one walking in front of the other, on their walking gaits has not been investigated yet.

**Fig 1:**
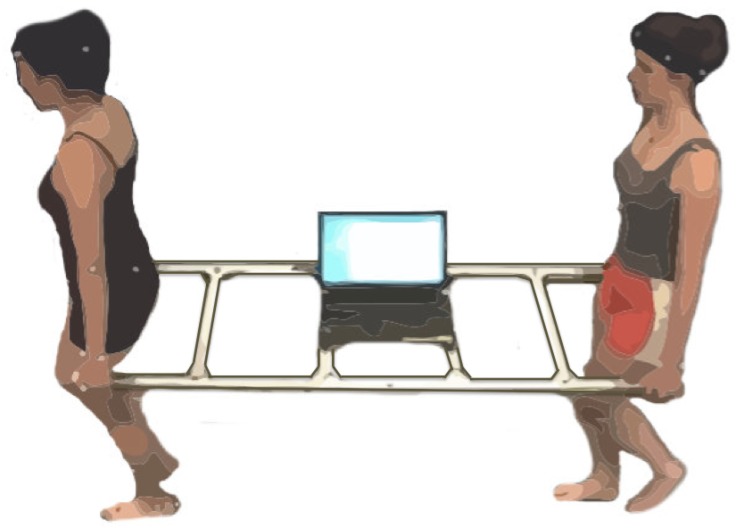
Paired trial. Two subjects were asked to walk while mechanically paired through a stretcher-like object. Each subject was equipped by 34 reflective markers that were used in combination with a motion capture system (VICON) to track the kinematic movements. A computer, placed on the center of the stretcher-like object, was used to record the force data from four 3D force sensors integrated in each handle.

In this paper, we will present a particular case of interpersonal coordination that occurs when two persons walk while physically paired by carrying an object together (see Fig 1). This configuration may be associated to quadrupedal locomotion.

The aim of this work is to investigate the implication of the coupling on the walking gaits of each human subject and understand the mechanism of gait synchronization by studying a simple model consisting of two bipedal agents connected with a spring-damper. Through such a study the following questions will be investigated:

- Do humans alter their gaits when they are physically paired with another human through an object while one is walking in front of the other?
- Do humans temporally coordinate their gaits when they are physically paired? If yes, do they exhibit typical “quadrupedal” gaits, and if so, which ones?
- To what extent the coordination is caused by the passive mechanical connection of the two bipedal systems?
- Is it possible to replicate the experimental data with a paired Spring Loaded Inverted Pendulums (SLIPs) model?
- Is it possible to predict the resulting paired walking gait after fitting each pendulum (SLIP) separately with data from the free (not paired) walking experiments?

The rest of this paper is organized as follows. In Section 2 the results of human experiments will be presented: Center of Mass (CoM) displacement, CoM velocity, Gait Cycle Time (GCT), and step length in unconstrained walking and mechanically paired walking with another subject will be compared, and gait foot-fall patterns of the two paired bipeds will be analyzed. In Section 3 a simple model of mechanically paired bipeds will be presented. The systematic parameter search for finding periodic orbits of this mechanical model will be described in Section 4. Finally, in Section 5 a comparison between the experimental data and simulation results will be presented.

## 2 Human-human interactive locomotion: Experiments

In this section, we present and analyze the kinematic data collected in the paired-walking experiments, where two subjects carry a stretcher-like object together (see Fig 1).

### 2.1 Methods

The experiments were conducted at gait laboratory of the Centre Hospitalier Universitaire Vaudois (CHUV), Lausanne, Switzerland, according to the principles expressed in the Declaration of Helsinki. Participants provided their written consent on the experiment protocol approved by the EPFL human research ethics committee (HREC No 004-2015) (see S1 Ethic Statement). Six healthy subjects with a mean age of 27 participated in the experiments. More details about the subjects’ anthropometrics data can be found in Table 1.

**Table 1:** Anthropometric data for each subject. Leg length was measured between the marker placed on the hip and the one placed on the ankle.

Subject ID	Age	Weight	Height	Leg Length	Gender
1	25	88 Kg	1.84 m	0.99 m	Male
2	26	70 Kg	1.74 m	0.95 m	Male
3	28	80 Kg	1.80 m	0.94 m	Male
4	34	64 Kg	1.78 m	0.95 m	Male
5	31	57.5 Kg	1.65 m	0.88 m	Female
6	21	61 Kg	1.64 m	0.78 m	Female

As Table 2 shows, the same subjects were used to compose different pairs and perform different roles (i.e., walking in front (A) or behind (B) the object).

**Table 2:** For each pair, the corresponding subjects are reported with respect to their specific position within the pair with the convention that Subject A is walking in front of the stretcher-like object, and Subject B is walking behind it.

Pair ID	Subject A (in front)	Subject B (behind)
01	Subject 1	Subject 2
02	Subject 3	Subject 4
03	Subject 4	Subject 3
04	Subject 5	Subject 3
05	Subject 3	Subject 5
06	Subject 6	Subject 5
07	Subject 5	Subject 6

Each experiment consisted of two trials:

- Solo trial
- Paired trial In the first case, the subjects were asked to walk for 12 m two times, back and forth. Each of these sequences will be indicated as a ‘Trial’; as a result, for each pair of subjects we will have 4 trials. In the second case, the same subjects were asked to walk two times while paired by a stretcher like object with a total weight of 8 kg and a length of 150 cm.

The average number of steps for a subject changes depending on the type of trial: on average, a solo trial consists of 8.8 ± 1.06 number of steps per subject per trial, while the average number of steps in a paired trial is 8.12 ± 0.49.

Overall, 7 pairs, made by a combination of 6 different subjects, performed the trials. A motion capture system (VICON) was used to track the kinematic movements of each subject with 34 markers placed on the subject’s body according to the Plug-In-Gait convention [5]. For the off-line data processing we used two commercial software packages: the Vicon Nexus software and MATLAB (The MathWorks Inc.). Nexus software was used to label markers, detect gaps in the data and filter them through a low-pass digital Butterworth filter that filters out signal noise above 300 Hz. Finally, MATLAB scripts were used to compute all the gait parameters of interest, described below:

#### Subjects’ CoM trajectories

Anthropometric tables [6] are used to compute the CoM position over time for each subject. In this way we can analyze the motion of the CoM along the vertical direction for each subject in each pair and such a motion is compared between the solo and paired trials.

#### Subjects’ GCT

GCT is defined as the time interval between the same walking event, such as two consecutive contact events of the same foot (e.g., right or left) on the ground. Finding the local minima of the heel trajectory, we estimate the times at which the heel strike happens, and thus, we can compute the time difference between two consecutive heel strikes of the same foot. For each subject the mean GCT and the standard deviation are computed for all the solo and paired trials, and GCT variation among solo trials, paired trials in position A (front) and paired trials in position B (back) is investigated.

#### Subjects’ step length

Step length is defined as the distance between the heel position of one foot and that of the other foot at the heel strike moment. For each subject the mean step length is extracted from all the solo and paired trials. Subjects’ step length variation among solo trials, paired trials in position A (front) and paired trials in position B (back) is investigated.

#### Subjects’ CoM velocity

By differentiating the CoM trajectory with respect to time, we obtain the CoM forward velocity for each subject. For all subjects CoM forward velocity variation among solo trials, paired trials in position A (front) and paired trials in position B (back) is investigated. Neither vertical nor lateral velocity has been analyzed.

#### Gait synchronization

A pair is considered to have a synchronized gait when

- The subjects have the same stride durations,
- The phase lag between the two subjects is constant.

We assume that the first condition (i.e., same stride duration) is satisfied when the absolute difference between stride durations of the two subjects within a pair is less then 4% of their mean stride duration. Such a threshold was chosen to be comparable with the coefficient of variation of a healthy subject during a 9-min walk, as presented in [7]. Regarding the second condition for synchronization, in order to find the phase lag between the subjects, for each trial the heel trajectories are first cut into *n* time windows, where *n* is the number of strides. Then, the cross-correlation between the heel trajectory *x*(*t*) of Subject A and *y*(*t*) of Subject B in each time window (of length N) is computed according to the following formula:

$$\begin{array}{c} c\left(m\right)={\hat{R}}_{xy}\left(m-N\right),\;m=1,2,\ldots ,2N-1 \end{array}$$

where

$$\begin{array}{c} {\hat{R}}_{xy}\left(m\right)=\{\begin{array}{cc} \begin{array}{c} \sum_{n=0}^{N-m-1}x_{n+m}y_{n},\\ {\hat{R}}_{yx}\left(-m\right), \end{array} & \begin{array}{c} m\geq 0,\\ m<0. \end{array} \end{array} \end{array}$$

Once the maximum cross-correlation is found, the corresponding lag scaled to [0°, 360°] is denoted as the phase lag, *ϕ*. Intuitively, *ϕ* determines the shift between the two heel trajectories that maximizes the correlation between the signals. The phase lag is considered to be constant as long as its variation is within a tolerance of ±10°.

#### Interlimb coordination

Six markers are placed on the stretcher-like object in order to analyze its position over time. Moreover, in order to have a measure of the upper limb motion, we compute the relative displacement between the CoM of the stretcher-like object and the CoM of each subject in the forward direction. Finally, we try to find a correlation between this relative displacement and the step duration.

Repeated measures ANOVA is performed for each of the three dependent variables (GCT, step length, and forward velocity) with three different conditions (solo trial, paired trial in position A (front), and paired trial in position B (back)). A post-hoc multiple comparison (Tukey’s HSD test with alpha = 0.05) is then applied to all the significant results.

Moreover, a statistical shape analysis (Procrustes analysis [8–10]) is performed in order to compare the mean CoM trajectories in the vertical direction of the paired trials and those of the solo trials. Procrustes analysis is based on the evaluation of the difference between the shape of two curves (in our case, the CoM trajectory of the solo trial in the vertical direction and that of the paired trial, normalized to percent gait cycle and interpolated at 1000 points). After computing the sum of the squared distances (SSD) between the corresponding points of the two curves, the square root of such SSD can be used as a statistical measure of the difference in shape, called Dissimilarity Measure (DM) (Eq (1)),

$$DM=\left(\sqrt{{\left({\tilde{z}}_{1}\;-\;z_{1}\right)}^{2}+\cdots +{\left({\tilde{z}}_{n}\;-\;z_{n}\right)}^{2}}\right),$$ (1)

where ${\tilde{z}}_{i}$, *i* = 1, …, *n* is the coordinate of one point on the first curve while *z*_*i*_, *i* = 1, …, *n* is the coordinate of the corresponding point on the second curve.

Finally, Pearson correlation coefficient between arms’ oscillations period and step period is calculated in order to analyze the interlimb coordination.

### 2.2 Main results

In this subsection we will present the main results that were obtained from the experiments using the methodologies explained in Subsection 2.1.

#### 2.2.1 Subjects’ CoM trajectories

**Fig 2:**
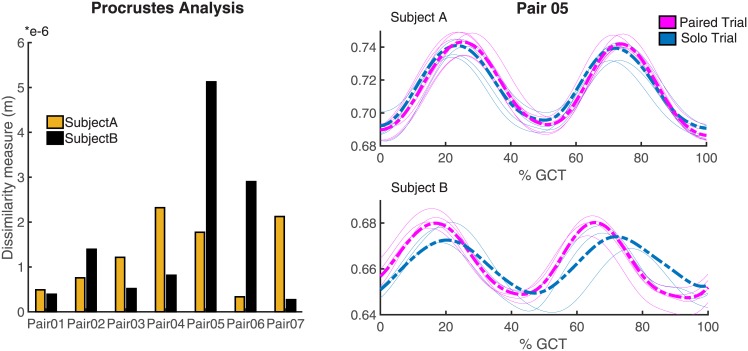
Procrustes analysis between the mean CoM trajectories of solo trial and paired trial for each pair. On the left, the dissimilarity measure obtained from a comparison between the mean CoM trajectories of solo trial and paired trial for each subject in the vertical direction is presented. For each pair, the yellow bar refers to the CoM trajectory of Subject A while the black bar is associated to Subject B. On the right, one example for the ‘furthest match’ result (Pair05) is shown.

Fig 2 shows Procrustes analysis results for the mean CoM trajectories in the vertical direction of the solo and paired trials for each pair. According to the DM value (see Eq (1)), we define the ‘closest match’ result as the one for which the DM is lowest and the ‘furthest match’ result as the one with the highest DM value. The furthest match results between the CoM vertical displacement of the solo trial and paired trial are reported in Fig 2, on the right.

As shown in Fig 2, the vertical CoM displacement of each subject is not affected by the mechanical coupling (at least for the 8 kg object used for the experiments).

#### 2.2.2 Subjects’ GCT

**Fig 3:**
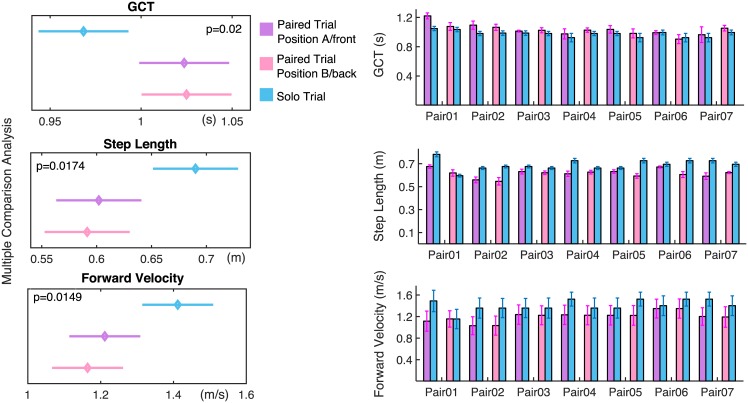
Statistical analysis for subjects’ GCT, step length and CoM velocity. Plots on the left show the post-hoc multiple comparison analysis (Tukey’s HSD test with alpha = 0.05). Such a comparison is applied to all the significant results of repeated measures ANOVA performed for three dependent variables (GCT on top, step length in the middle and forward velocity on bottom) with three conditions: solo trial (blue bars), paired trial when the subject is in front (violet bars) or back (pink bars) with respect to the stretcher-like object. On the right, for each dependent variable, the mean values and standard deviations over all the solo (blue bars) and paired (pink and violet bars) trials for each subject within a pair are shown.

On the top-left of Fig 3 it is shown that there is a significant tendency (*p* = 0.02) for each subject to increase the GCT when paired compared to the solo case; this increase on average is 0.12 s. Moreover, once paired, there is a statistically negligible difference of GCT when the subject is in position A (e.g., in front of the stretcher like object) or in position B (e.g., behind the stretcher like object). These results are summarized in Table 3, which reports Tukey HSD test results. Moreover, on the top-right of Fig 3, the overall GCT’s mean and standard deviation for each subject within a pair during the solo and paired trials are presented. From the previous plot it is also possible to notice that during the paired trials, subjects within a pair have almost the same GCT; in particular, on average, the GCT difference between subjects A and subjects B is 0.024 ± 0.068 *s*, which is less than 2% of the average GCT in all trials.

**Table 3:** Results of Tukey HSD test for three dependent variables (GCT, step length and forward velocity) with conditions 1 (solo trial), 2 (paired trial in position A (front)), and 3 (paired trial in position B (back)). For each dependent variable the average between conditions *I* and *J*, with *I*, *J* = 1, 2, 3 with I≠J is denoted by ${\bar{x}}_{I}-{\bar{x}}_{J}$. The asterisk indicates which conditions statistically differ (*p* < 0.05).

Dependent Variables	Conditions	${\bar{x}}_{I}-{\bar{x}}_{J}$	Lower Limit	Upper Limit	p
GCT	1, 2*	−0.055	−0.1047	−0.0059	0.03
	1, 3*	−0.056	−0.1060	−0.0072	0.03
	2, 3*	−0.001	−0.0507	0.0481	0.99
Step Length	1, 2*	0.088	0.010	0.165	0.03
	1, 3*	0.098	0.021	0.176	0.02
	2, 3	0.010	−0.066	0.088	0.90
Forward Velocity	1, 2*	0.2	0.005	0.395	0.04
	1, 3*	0.247	0.052	0.442	0.02
	2, 3	0.047	−0.147	0.242	0.74

#### 2.2.3 Subjects’ step length

In the middle-left of Fig 3 it is shown that, compared to the solo case, once paired, the step length decreases by 7.62 cm on average (*p* = 0.0174). Moreover, the difference between step lengths of each subject in position A (front) and position B (back) are statistically negligible. These results are summarized in Table 3, which reports Tukey HSD test results. An overall evaluation of the step length mean and standard deviation during the solo and paired trials is also reported on the middle-right of Fig 3. The latter plot can be used to evaluate how the step lengths of subjects within a pair converge to similar values; on average, the step length difference between subjects A and subjects B is 0.023 ± 0.035 *m* which is 4% of the average step length in all trials.

#### 2.2.4 Subjects’ CoM velocity

The previous results in terms of the increase in the GCT and reduction of the stride length, when paired, were also supported by the CoM velocity analysis.

From the bottom-left of Fig 3, we notice a statistically significant (*p* = 0.0149) reduction (by 0.24 *m*/*s* on average) in the forward walking speed as a consequence of the coupling. Also in this case subjects have statistically negligible different velocities when they are in position A (front) or position B (back).These results are summarized in Table 3, which reports Tukey HSD test results. Moreover, for each subject within a pair, the overall mean and standard deviation of the forward velocity are shown, both for solo and paired trials (see Fig 3, on the bottom-right). From the previous plot it is possible to evaluate how subjects within a pair agree in terms of the forward velocity: the average difference between the CoM forward velocities of subjects A and subjects B is very small ($0.005\pm 0.0127\;\frac{m}{s}$).

#### 2.2.5 Gait synchronization

**Fig 4:**
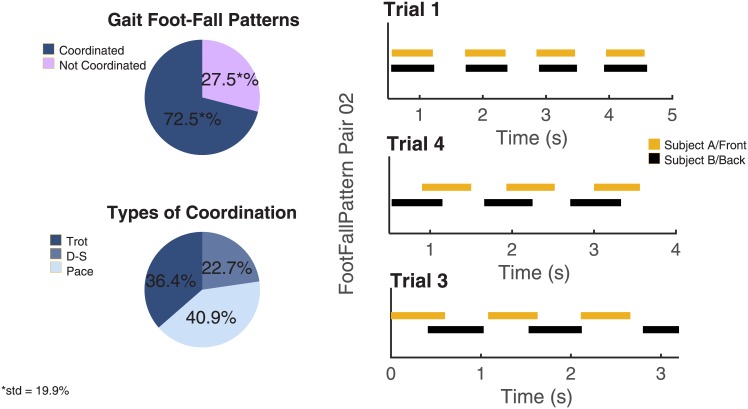
Percentage of synchronization and corresponding types of the resulting gait. The plot on the top-left shows the percentage of the paired trials with coordinated gaits versus those with not coordinated gaits. The plot on the bottom-left shows the percentage of different types of quadrupedal-like gaits (e.g., trot, D-S, and pace) among all of the coordinated gaits. On the right an example of gait foot-fall pattern of Pair02 at three different trials is reported. Here each segment represents the stance foot (the one on the ground) of one subject. Yellow segments represent the right foot of Subject A while the black segments represent the right foot of Subject B. Given the symmetry between left and right foot-fall patterns, only one foot is represented for each subject.

Subjects within a pair tend to converge to the same GCT, as explained in Subsection 2.2.2. Therefore, by definition of a synchronized gait as presented in Subsection 2.1, in order to examine synchronization, phase lag needs to be analyzed. Based on our phase lag estimation, as shown on the top left of Fig 4, on average, the subjects exhibit a synchronized gait in 72.5 ± 19.9% of each trial.

Moreover, if we consider the original system, which is made up of an object and two subjects connected, as a single body with four legs, we observe that some patterns, which can be generally associated to quadrupedal gaits, such as ‘Diagonal-Sequence’, ‘Pace’, or ‘Trot’ [11], can be associated with the emerging synchronized gaits (see Fig 4, on the right).

In ‘Diagonal-Sequence’ gait, two limbs alternate with three limbs in supporting the weight of the system. Thus, eight different combinations of limb support occur during one stride: four combinations of three-leg support interspersed with four combinations of alternating two-leg support (right/left diagonal and right/left lateral support). In the ‘Pace’ gait the two lateral limbs are used alternately for weight support. For the ‘Trot’ gait, right and left diagonals alternate in supporting the weight.

The phase lag *ϕ*, as defined in Subsection 2.1, can be used to classify the quadrupedal-like gaits. More specifically, if *ϕ* ≈ 0° the two subjects converge to a pace-like gait while if *ϕ* ≈ 180°, the two subjects converge to a trot-like gait, and finally if *ϕ* ≈ 225° a diagonal sequence-like gait appears.

With the above definitions, among all the synchronized gaits pace gait (40.9%), trot gait (36.4%) and diagonal-sequence (D-S) (22.7%) can be identified (see the bottom-left of Fig 4).

#### 2.2.6 Interlimb coordination

**Fig 5:**
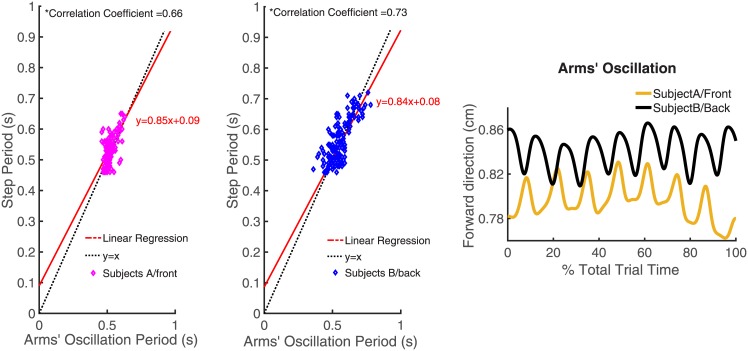
Interlimb coordination of Subject A/Subject B for each trial. The first two plots from the left show the relationship between arms’ oscillation periods and step periods in each paired trial and for each subject (Subject A, on the left, and Subject B, on the right). Correlation coefficients for each plot show that such a relationship between the aforementioned periods is linear (see black straight line obtained by a linear regression fitting) and that it is close to the line *y* = *x* (red line in the plots). The last plot on the right shows the relative displacement on the forward direction between the subjects’ (Subject A and B are represented in yellow and black, respectively) and object’s CoMs.

The interlimb coordination is presented here as the correlation between subjects’ step periods and the arms’ oscillation periods during paired trials. Arms’ oscillation is defined as the relative displacement over time between each subject’s CoM and the object’s CoM, computed along the forward direction. From such a displacement, which is almost periodic (see Fig 5, plot on the right), it is possible to extract the oscillation period for Subject A and B in each pair as the time difference between two local maxima (or two local minima) of the horizontal relative displacement between one subject’s and object’s CoM. The plot on the left in Fig 5 shows that the period of arms’ oscillation is almost equal to the step period. In this plot each point represents a pair step period-arms’ oscillation period for each Subject A and Subject B within a pair during paired trials. For Subjects on the back there is a larger dispersion of points that can be associated to the greater range of arms’ motions in the forward direction compared to the backward direction.

## 3 The model

Several models can be used for bipedal locomotion, however, perhaps the simplest model that can explain some key features of human walking, such as CoM displacement and Ground Reaction Forces (GRF), is the SLIP model, initially designed to model running but then extended to model walking as well [12, 13]. Because of its simplicity and explanatory power we decided to use such a model as a constituent unit to describe two bipedal agents walking together while mechanically paired.

**Fig 6:**
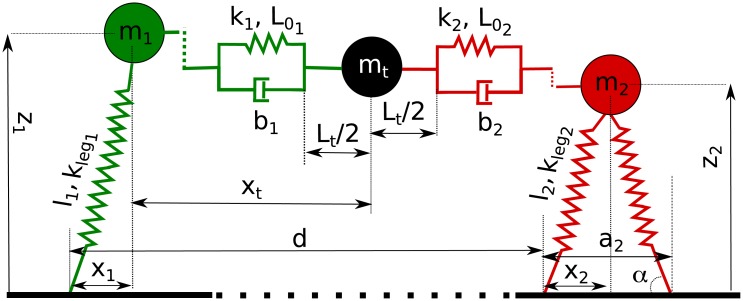
Paired SLIPs model. On the left one SLIP is in the single support phase (the swing leg is not represented), while the SLIP on the right is in the double support phase. In the middle a mass is connected to the CoM of each pendulum through a spring and damper system representing the arms’ impedance. We indicate the center of mass of each SLIP by *m*_*i*_ for *i* = 1, 2, while *l*_*i*_, $k_{leg_{i}}$ and *α*_*i*_ represent the SLIPs’ initial leg length, the leg stiffness, and the angles of attack, respectively; *k*_*i*_, *b*_*i*_ and $L_{0_{i}}$ are the impedance parameters and the equilibrium lengths of each connection between the SLIP’s CoM and the CoM of the object, which has a total length of *L*_*t*_ and its relative position with respect to the first SLIP is indicated as *x*_*t*_. Finally, *x*_*i*_ and *z*_*i*_ indicate each SLIP’s CoM horizontal and vertical position while *d* is the distance between the foot point of the first pendulum’s stance spring and that of the second pendulum.

As shown in Fig 6, in our model, two SLIP models are linked together with a spring-mass-damper connection, where the following assumptions are made:

- Double support phase is included;
- The mass of each agent is concentrated at its CoM;
- The model has point feet;
- Arms effect in the coupling is represented as a spring and damper system with constant impedance;
- The vertical interaction forces between the subjects and the object does not affect the walking gaits of the subjects.

The first three points are directly related to the choice of using the SLIP model as a constituent unit, while the final two points refer to the coupling between the two bipedal agents. The assumption of using a spring and damper system to model the coupling can be associated on one side to the mechanical characteristics of human muscles and on the other side to the arms’ oscillatory behaviour observed from the experimental data. Finally, the last assumption is related to a qualitative analysis of the interaction force data, where in the experiments, by using Optoforce sensors integrated in each handle of the stretcher-like object, we observed that the variations of the force in the forward direction result to be greater than the vertical ones (see for example S2 Fig).

### 3.1 SLIP model

The SLIP model is one of the simplest models that yields human-like results in terms of walking behaviour [13]. The SLIP model can be used to model both walking and running [12, 13]. As previously mentioned, with the SLIP model it is possible to reproduce the GRFs that point towards the CoM and depend only on the leg length and stiffness. In such a model, in order to be able to replicate human data, it is necessary to set appropriate values for the parameters such as spring stiffness, angle of attack, and mass. Moreover, the only variables of the model that have to be initialized with appropriate values are the CoM position at the apex (that is, the point where the height of the CoM reaches its maximum value) and the CoM velocity or, alternatively, the initial energy of the system. To avoid dependency of the vertical position on the leg length, the vertical position of the apex is always defined with respect to the one reached by the CoM when the leg’s spring is at rest. The horizontal position at the apex is computed with respect to the support point.

### 3.2 Paired SLIPs model

The paired model is made up of two SLIPs connected together by a parallel mass, spring and damper system, as shown in Fig 6. This system can be described by the following equations of motion

$$\begin{array}{cc} m_{1}{\ddot{x}}_{1} & =(P_{1}x_{1})\cdot \gamma -Q_{1}(a_{1}-x_{1})\cdot \beta -F_{1t}, \end{array}$$ (2)

$$\begin{array}{cc} m_{2}{\ddot{x}}_{2} & =(P_{2}x_{2})\cdot \gamma -Q_{2}(a_{2}-x_{2})\cdot \beta -F_{2t}, \end{array}$$ (3)

$$\begin{array}{cc} m_{1}{\ddot{z}}_{1} & =(P_{1}z_{1})\cdot \gamma -Q_{1}z_{1}\cdot \beta -m_{1}g, \end{array}$$ (4)

$$\begin{array}{cc} m_{2}{\ddot{z}}_{2} & =(P_{2}z_{2})\cdot \gamma -Q_{2}z_{2}\cdot \beta -m_{2}g, \end{array}$$ (5)

$$\begin{array}{cc} m_{t}({\ddot{x}}_{1}+{\ddot{x}}_{t}) & =F_{1t}+F_{2t}, \end{array}$$ (6)

where

- *x*_*i*_, ${\dot{x}}_{i},$ and ${\ddot{x}}_{i}$ are respectively the absolute position, velocity, and acceleration along the forward direction, of pendulum *i* with *i* = 1, 2;
- *z*_*i*_, ${\dot{z}}_{i},$ and ${\ddot{z}}_{i}$ are respectively the absolute position, velocity, and acceleration along the vertical direction, of pendulum *i*, with *i* = 1, 2;
- *x*_*t*_, ${\dot{x}}_{t},$ and ${\ddot{x}}_{t}$ are respectively the relative position, velocity, and acceleration along the forward direction, of the carried mass *m*_*t*_;
- $P_{i}=k(\frac{l_{0i}}{\sqrt{x_{i}^{2}+z_{i}^{2}}}-1)$ for *i* = 1, 2 and *l*_0*i*_ represents the rest length of the leg spring of pendulum *i*;
- $Q_{i}=k\left(\frac{l_{0i}}{\sqrt{{\left(a_{i}-x_{i}\right)}^{2}+z_{i}}}-1\right)$ for *i* = 1, 2 and *l*_0*i*_ represents the rest length of the leg spring of pendulum *i*;
- $F_{1t}=-k_{1}(L_{1}-L_{0_{1}})-b_{1}{\dot{L}}_{1}$ is the force applied by the first pendulum to the object;
- $F_{2t}=k_{2}(L_{2}-L_{0_{2}})+b_{2}{\dot{L}}_{2}$ is the force applied by the second pendulum to the object;
- $L_{1}=x_{t}-\frac{L_{t}}{2}$ is the horizontal distance between the mass of the first pendulum and the object;
- *L*_01_ is the equilibrium horizontal distance between the mass of the first pendulum and the object;
- $L_{2}=(d+x_{2})-((x_{1}+x_{t})+\frac{L_{t}}{2})$ is the horizontal distance between the mass of the second pendulum and the object;
- *L*_02_ is the equilibrium horizontal distance between the mass of the second pendulum and the object;
- *L*_*t*_ is the total length of the object;
- *g* is the gravity acceleration;
- *m*_1_, *m*_2_, and *m*_*t*_ are respectively the masses of the pendulum that is behind the carried object (with respect to the direction of motion), the pendulum that is in front, and the carried object;
- *d* is the distance between the horizontal foot point of the first pendulum’s stance spring and that of the second pendulum;
- *a*_*i*_ is the distance between the stance footpoints of the pendulum *i*(*i* = 1, 2);
- *γ*, *β* *ϵ*{0, 1} are two constants used to switch among three main walking states, as follows:
  $$\{\begin{array}{lll} \gamma =1 & \beta =0 & \text{single support after reaching the apex}\\ \gamma =1 & \beta =1 & \text{double support}\\ \gamma =0 & \beta =1 & \text{single support before reaching the apex}. \end{array}$$

For this model, the initial conditions are crucial; in particular, it is necessary to define

- $x_{apex_{i}}\left(0\right)$, *i* = 1, 2, which is the horizontal position of each pendulum’s CoM at the apex with respect to the foot position;
- $z_{apex_{i}}\left(0\right)$, *i* = 1, 2, which is the vertical CoM position of each pendulum at the apex height that is defined with respect to the CoM vertical position at rest;
- *E*_0*i*_, *i* = 1, 2, which is the initial energy of each pendulum.

Furthermore, in order to better replicate human gait, we choose the range of the following parameters according to [6, 13]:

- Masses (*m*_1_, *m*_2_) and initial leg lengths (*l*_01_, *l*_02_) of each pendulum
- Legs’ stiffness (*k*_*leg*_) and angle of attack (*α*)
- Arms’ stiffness (*k*_1_, *k*_2_) and damping (*b*_1_, *b*_2_)

Table 4 shows the mechanical range of these parameters.

**Table 4:** Ranges of parameters and initial conditions used to evaluate the model’s behaviour. The masses range, both for the table and the pendulums, and the length of the object and the pendulums’ legs can be easily set according to the experimental parameters (see Table 1). Legs’ stiffness, angle of attack and initial energy can be set as described in [13]. Finally, arms’ impedance range has been selected according to [14].

*m*_*1,2*_ (kg)	*l*_*1,2*_ (m)	m_*t*_ (kg)	*L*_*t*_(m)	*k*_*leg*_(N/m)	α(rad)	*k*_*1,2*_(N/m)	$b_{1,2}\left(\frac{\text{Ns}}{m}\right)$
[70, 90]	[0.7, 1.0]	7.5	2	[1.2 ⋅ 10^4^, 2.2 ⋅ 10^4^]	[1.16, 1.36]	[5 ⋅ 10^2^, 8 ⋅ 10^3^]	[40, 325]

Regarding the arm equilibrium lengths (*L*_01_ and *L*_02_), we note that since we always assume that at the beginning of the simulations the arms’ lengths (*L*_1_(0) and *L*_2_(0)) are equal to their equilibrium lengths, and the only way *L*_01_ and *L*_02_ appear in the equations of motion is in the form of (*L*_1_ − *L*_01_) and (*L*_2_ − *L*_02_), the specific values of *L*_01_ and *L*_02_ will not effect the results.

Since the paired-SLIPs model described above has no means of injection of energy, but has dissipation of energy, it generally does not possess periodic solutions. However, as we can see in Section 4 and 5, it is possible to find approximate periodic orbits up to a very small threshold (e.g., state values that vary less than 0.1% from step to step) in a relatively large window of time (100 *s*). With this definition of an approximate periodic orbit, we are still able to study the role of passive mechanism in achieving synchronized periodic solutions in paired walking.

To obtain periodic solutions as defined above, for given m_*i*_, *l*_*i*_, and initial conditions $z_{apex_{i}}\left(0\right)$, and initial energy, $E_{0_{i}}$, by a systematic search (Subsection 4.1), we find legs’ stiffness ($k_{leg_{i}}$), angle of attack (*α*_*i*_), arms’ stiffness (*k*_*i*_), and damping (*b*_*i*_) such that the paired SLIP model reaches a periodic walking gait as previously defined.

## 4 Periodic solutions of the model

### 4.1 Systematic search

In this section we present a method to find the system parameters (arms/legs stiffness and angles of attack) which lead to solutions that respect the approximate periodic condition. Because of the complexity of the model and the high dimensionality of the search grid (*N*^*P*^, where *P* is the number of parameters to analyze), the analysis is completed in three steps such that in each step we only search for two parameters.

In each step of the systematic search we evaluate the following *Periodicity* *Score* (*S*_*P*_) function:

$$\begin{array}{c} S_{P}=log_{10}(\frac{\xi }{RMS}), \end{array}$$

where *ξ* (∼10^−3^) is the maximum variation between two consecutive apexes, that is,

$$\begin{array}{c} |{\left(x_{{\text{apex}}_{k}},z_{{\text{apex}}_{k}}\right)}_{i+1}-{\left(x_{{\text{apex}}_{k}},z_{{\text{apex}}_{k}}\right)}_{i}|<\xi , \end{array}$$

for *k* = 1, 2. The value of the tolerance *ξ* has been set according to the variation of the apex height of the subjects during the experiments. Finally, $RMS=\sqrt{RMS_{1}^{2}+RMS_{2}^{2}}$, where *RMS*_*k*_, with *k* = 1, 2, is the root mean square of the norm of the difference between subsequent apex positions of pendulum *k*; that is,

$$\begin{array}{c} RMS_{k}=\sqrt{\frac{1}{n-j}\overset{n-1}{\underset{i=j}{\sum }}{\left|{\left(x_{{\text{apex}}_{k}},z_{{\text{apex}}_{k}}\right)}_{i+1}-{\left(x_{{\text{apex}}_{k}},z_{{\text{apex}}_{k}}\right)}_{i}\right|}^{2}},\;k=1,2 \end{array}$$

In this formula,

- The index *n* is the total number of simulated steps, considering a maximum time of 100 *s*;
- The index *j* is the smallest integer for which $|{\left(x_{{\text{apex}}_{k}},z_{{\text{apex}}_{k}}\right)}_{i+1}-{\left(x_{{\text{apex}}_{k}},z_{{\text{apex}}_{k}}\right)}_{i}|<\xi$ holds for all *i* > *j*; that is, for indices greater than *j* the difference between two consecutive apex points are always less than the defined tolerance. If *j* does not exist, the gait is considered to be not periodic, and its score is assigned as 0.

The periodicity score described above is defined based on the notion of the ‘return map’, which sends the horizontal and vertical positions of each pendulum at the apex in the current step to the ones in the next step. Intuitively, the higher the periodicity score is, the closer is the apex to its value in the previous steps. In this way we are able to define a score that is capable of measuring the periodicity of the solutions for our system.

The three steps of the systematic search are explained in details in the following:

- Step 1—Unpaired pendulums: Based on the periodicity score *S*_*P*_, the best value of the leg stiffness and the angle of attack is found for the single bipedal agent (SLIP);
- Step 2—Paired system: Arms’ impedance (which is assumed to be the same for both pendulums) that maximizes *S*_*P*_ is found. Here, the angle of attack and the leg stiffness are fixed to the values found in Step 1.
- Step 3—Paired system: Best values of the leg stiffness and the angle of attack for each pendulum are found according to the periodicity score. Arms impedance is fixed to the value found in Step 2.

An alternative approach could be searching for all four parameters (i.e., leg stiffness and angle of attack, arm stiffness and damping) at the same time, however, with the above systematic search, at each stage only two parameters are searched for; this approach greatly reduces the computation time.

We note that the first two steps of the systematic search are important to find the legs and arms impedance able to guarantee a periodic walking behaviour of the paired system as defined in Subsection 3.2: at the beginning, the two pendulums are unpaired, and we look for individual locomotion periodicity by testing different leg stiffnesses and angles of attack; then the paired system is analyzed by fixing the legs stiffness and angles of attack and testing the walking gait for different arms impedance. Finally, Step 3 is necessary to better adjust legs stiffness and angles of attack of each pendulum while paired compared to the values found in Step 1, which were optimized for the unpaired system.

**Fig 7:**
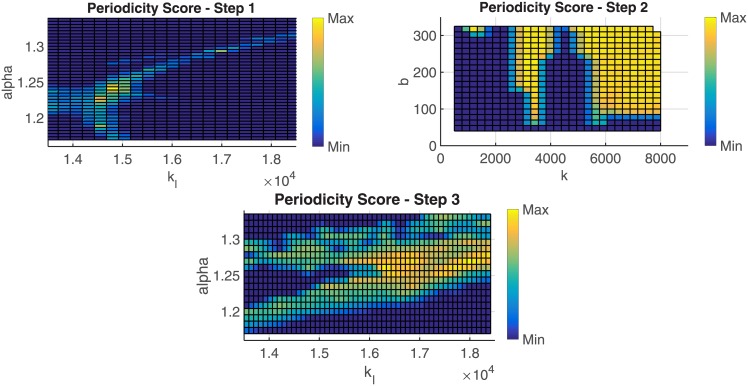
Systematic search. The figure shows the three main steps of the systematic search: in the first step (plot on the top-left) the best angle of attack (*α*) and leg stiffness (*k*_*l*_) for each pendulum separately (no coupling) are found. In the step 2 (plot on the top-right) the best arms’ stiffness parameters (spring stiffness *k* and damping parameter, *b*) for each pendulum in the paired system are found. Finally, in step 3 (plot on the bottom) the best angle of attack (*α*) and leg stiffness (*k*_*l*_) for each pendulum while paired are found. Here, the arms’ stiffness and damping are fixed (*k* = 3.5 ⋅ 10^3^ N/m, *b* = 320 Ns/m) according to the best solution found in the previous step of the systematic search. In all cases yellow regions indicate pairs of parameters that guarantee a high periodicity score while the blue regions represent pairs of parameters that result in a low periodicity score.

Fig 7 presents the results of the three steps of the algorithm. It turns out that the system has a higher periodicity score for higher stiffness values.

### 4.2 An example of a synchronized gait

**Fig 8:**
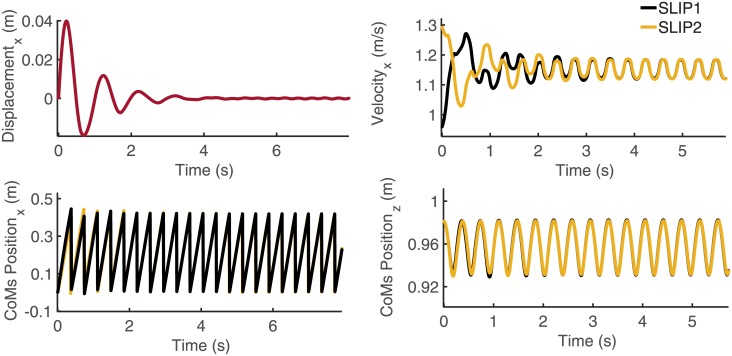
CoMs positions and velocities versus time in the synchronized gait. Results obtained through the systematic search are shown. In the top-left graph, the relative displacement, in the forward direction, between the two CoMs is presented. In the bottom-left graph, the yellow curve refers to the first pendulum’s CoM displacement in the forward direction while the black curve represents the other pendulum’s CoM displacement. In the top-right graph, the black curve refers to the forward velocity of the first pendulum while the yellow curve refers to that of the second pendulum. Finally, in the bottom-left graph, the CoM vertical displacement is presented for both pendulums.

Since in a periodic gait, as defined in Subsection 3.2, the trajectories and the velocities of the two pendulums are almost identical (with a tolerance of 10^−3^), the periodic gaits found by the systematic search are necessarily synchronized gaits as defined in Section 2. Here, we present an example of such a synchronized gait found by the systematic search algorithm (see Fig 8 and S1 Video). In this simulation, the mass and leg length are assumed to be the same for both pendulums (m_1_ = m_2_ = 80 kg, *l*_1_ = *l*_2_ = 1 m). With the initial conditions shown in Table 5, the parameters are found using the systematic search method, the outcome of which is shown in Table 6.

**Table 5:** Initial conditions in terms of energy, horizontal and vertical positions of the pendulums. Contrary to Step 1 and Step 3, different initial conditions are selected in Step 2, where the two SLIPs are paired, and we look for arms’ impedance that maximizes *S*_*P*_. Such a choice is important since we can allow the system to have dynamic interactions and to check whether, when starting with different conditions, the two modeled agents can reach a synchronized gait.

	Energy_1_	Energy_2_	*x*_*apex*_(0)	*z*_*apex*_(0)
Step 1	825 J	825 J	0	2.5 cm
Step 2	810 J	840 J	0	2.5 cm
Step 3	825 J	825 J	0	2.5 cm

**Table 6:** The set of parameters found by the systematic search algorithm. Such parameters guarantee periodicity of the solution of the paired-SLIPs model.

$k_{leg_{1,2}}$ (N/m)	*α*_1,2_ (deg)	$k_{arm_{1,2}}$ (N/m)	$b_{arm_{1,2}}$ (Ns/m)
17.9 ⋅ 10^3^	72.4	3100	200

As expected from the systematic search, the convergence of the system to a synchronized gait strongly depends on the right choice of parameters. By choosing a wrong set of parameters, the two SLIPs that start with different initial conditions will fall after a few steps (see S2 Video). This could suggest that once coupled, each simulated bipedal agent has to properly adjust its arms impedance and legs stiffness according to the behaviour of the other agent.

## 5 Data comparison

As explained in the Section 1, we would like to understand whether the behaviour of two subjects walking together while mechanically paired could be explained just by the passive mechanics of the overall system. To this end, the paired SLIPs model which is introduced in Section 3, is used in this section to replicate and predict the human experimental data. For each bipedal agent we use anthropometric data to set the mass and leg length for data comparison. With this comparison two main questions have to be addressed:

1. Is the model capable of reproducing the behaviour of the real paired system?
2. How well can the model predict the behaviour of the real paired system?

There is a slight difference between these two questions. In the first one, we want to force the model to capture the experimental data from the paired trials, while in the second one, just by using the knowledge of the preferred walking gait parameters of each subject in solo trial, we would like to check whether the model can predict the paired behaviour. To answer both aforementioned questions, two different data fitting analyses are proposed in Subsections 5.1 and 5.2.

### 5.1 Simulation of the paired behaviour by fitting the experimental paired trial data

In this subsection we present a method to examine whether the model is capable of reproducing the behaviour of the real paired system. To this end, we search for sets of model parameters that allow the system to reach a periodic walking gait as defined in Subsection 3.2 which is, at the same time, consistent with the experimental paired data; such a search for model parameters is referred to as ‘paired trial fitting’. To implement the ‘paired trial fitting’, it is necessary to introduce a new systematic search that is slightly different from the one described in Subsection 4.1. In particular, the three main steps of the systematic search in Subsection 4.1 remain the same, as well as the parameters we search for. However, since we want to constrain the model to fit the experimental paired trial data, a new score, called *Global* *Score* (*S*_*G*_), which will be evaluated at the end of each search step, is defined as follows:

$$\begin{array}{c} S_{G}=S_{S}\cdot S_{R}\cdot S_{P}, \end{array}$$

where *S*_*R*_ is the *Real* *Conditions* *Score* and is used to evaluate how close are the apex position and velocity of the modeled system to the experimental one, *S*_*S*_, is the *Synchronization* *Score*, which is defined based on the ratio of the GCT of Subject A to that of Subject B by which we measure how well synchronized the gaits are, and finally *S*_*P*_ is the score defined in Subsection 4.1. Exact definitions of *S*_*R*_ and *S*_*S*_ can be found in S1 Appendix.

In the simulations, the mass and leg length of each pendulum are set to be the same as those of the corresponding subject in the pair, while the mass of the object in between is set to be the same as that of the stretcher-like object from the experiments.

### 5.2 Simulation of the paired behaviour by fitting the experimental solo trial data

In order to evaluate the predictive power of the model presented in Section 3, the approach adopted here is to fit each modeled agent (that is, each SLIP) to each subject’s features, such as mass, leg length, initial velocity, apex height, etc. during the solo trial. We then search for sets of parameters (angle of attack, arms’ impedance, and legs’ stiffness) as described in Subsection 4.1; we call this search for the model parameters ‘solo trial fitting’. Unlike Subsection 5.1, no optimization for matching the simulations with the experimental data of the paired trial has been taken into account. The score used for the systematic search in this case is the one introduced in Subsection 4.1.

### 5.3 Methods and results

The implementations of the ‘solo trial fitting’ and the ‘paired trial fitting’ show that there exist solutions which respect the periodicity condition introduced in Subsection 3.2 for a specific set of impedance parameters. Several walking parameters of the paired model are compared with the experimental results in order to evaluate whether the model is able to replicate and/or predict humans’ behaviour.

In particular, the main evaluated variables are:

- CoM trajectory;
- GCT;
- Step length;
- Forward velocity;
- Gait foot-fall pattern.

We did not analyze the interlimb coordination since in the presented model the synchronized gait is obtained so that the interactions between the modeled agents and the object reduce over time in order to reduce energy dissipation.

**Fig 9:**
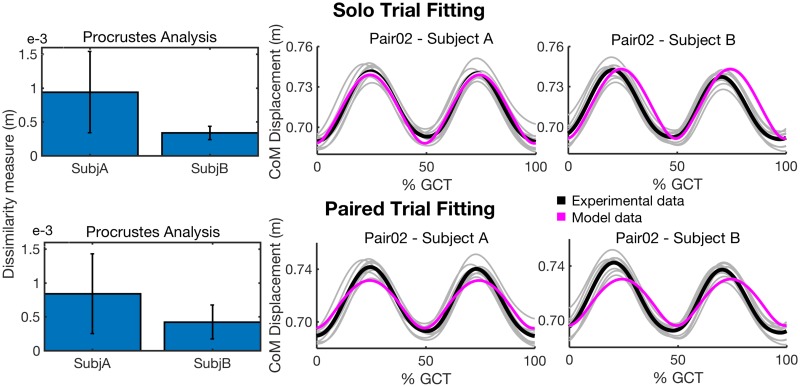
CoM vertical displacement comparisons. Procrustes analysis results are shown on the left for comparing CoM displacement of each human subject and the corresponding modeled agent in solo and paired trial fittings. An example of such a comparison is reported on the right for Pair02. The mean CoM vertical displacements of the real subjects are reported in black while the magenta curves represent the CoM vertical diplacements of the modeled agents.

With respect to the first parameter analyzed, the CoM vertical displacement of each modeled agent is compared with that of the corresponding subject. Moreover, the CoM displacement of each modeled agent is compared between the solo and paired trials through Procrustes analysis [10]. Fig 9 shows that in both solo and paired trial fittings the model is able to reproduce CoM vertical displacement of the subjects during paired trials. Moreover, in the solo trial fitting the model is able to reproduce human’s behaviour of keeping the same vertical CoM displacement between the solo and paired trials. Indeed, by applying Procrustes analysis [10] between each modeled agents’ CoM vertical displacement during the paired and solo trials, we obtained a dissimilarity measure close to zero in all the cases.

For the GCT, step length, and forward velocity, we performed repeated measure ANOVA with three different conditions, namely, solo trial, paired trial in position A (front), and paired trial in position B (back), as evaluated for the experiments (see Subsections 2.1).

**Fig 10:**
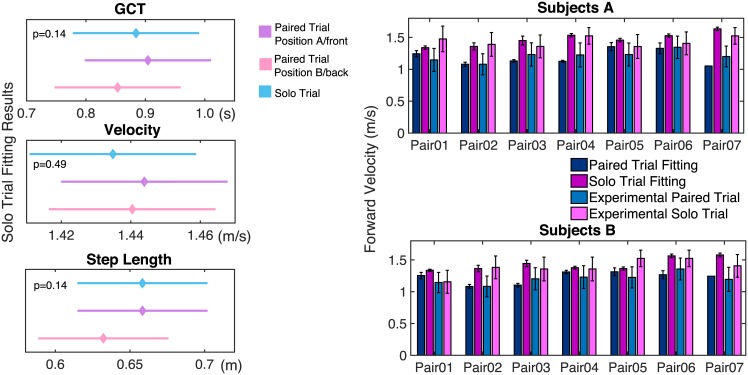
GCT, forward velocity and step length evaluations. On the left the output of a post-hoc multiple comparison analysis (Tukey’s HSD test with alpha = 0.05) is reported in order to clearly show the results of repeated measure ANOVA performed for three dependent variables (GCT on top, step length in the middle, and forward velocity on the bottom) with three conditions: solo trial (blue bars), paired trial when the modeled agent is in front (violet bars) and back (pink bars). The graphs on the right side show the means forward velocities and corresponding standard deviations for each pair for 4 different cases: paired trial fitting (dark blue bars), solo trial fitting (dark pink bars), experimental paired trial (light blue bars), and finally experimental solo trial (pink bars).

The graphs in Fig 10 show that, in contrast to human’s behaviour, in the solo trial fitting results there is not a significant tendency for each modeled agent to either increase the GCT or decrease the step length and the forward velocity when paired compared to the solo trial.

On the right side of Fig 10, we report the means and standard deviations of the forward velocities in four different cases: paired and solo trial fitting results and experimental solo and paired trials. We can observe that the forward velocity in the solo trial fitting is always higher than the one expected in the experimental paired trials. Indeed, the value of the forward velocity in solo trial fitting remains close to those of the experimental solo trials of each subject.

A final comparison between the behaviour of the paired SLIPs model and the experimental data is realized in terms of the gait foot-fall pattern. To perform such a comparison, just a short window of time of the experimental foot-fall pattern data can be evaluated because of the limitated size of the room where the experiments took place. As a consequence, the same time window will be considered in the comparison of the gait foot-fall pattern between the experiments and the simulations.

**Fig 11:**
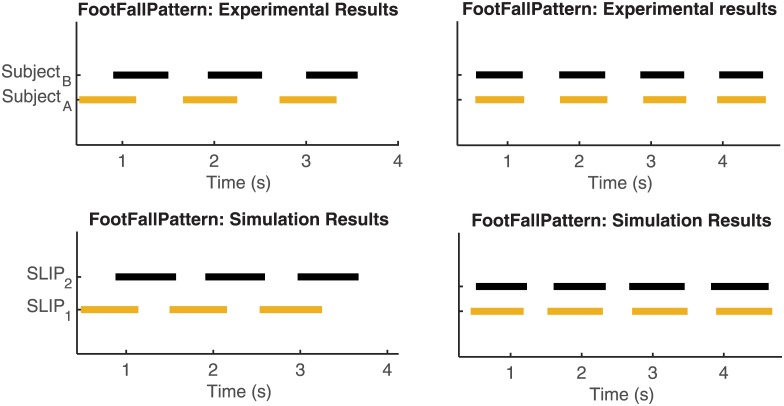
Foot-fall patterns comparison between the experimental data and simulation results (Pair 02, Trial 1 (left) and Trial 2 (right)). Simulation results obtained by ‘paired trial fitting’: In each plot yellow segments refer to the right foot of Subject A/pendulum in front, while black segments refer to the right foot of Subject B/pendulum behind.

**Fig 12:**
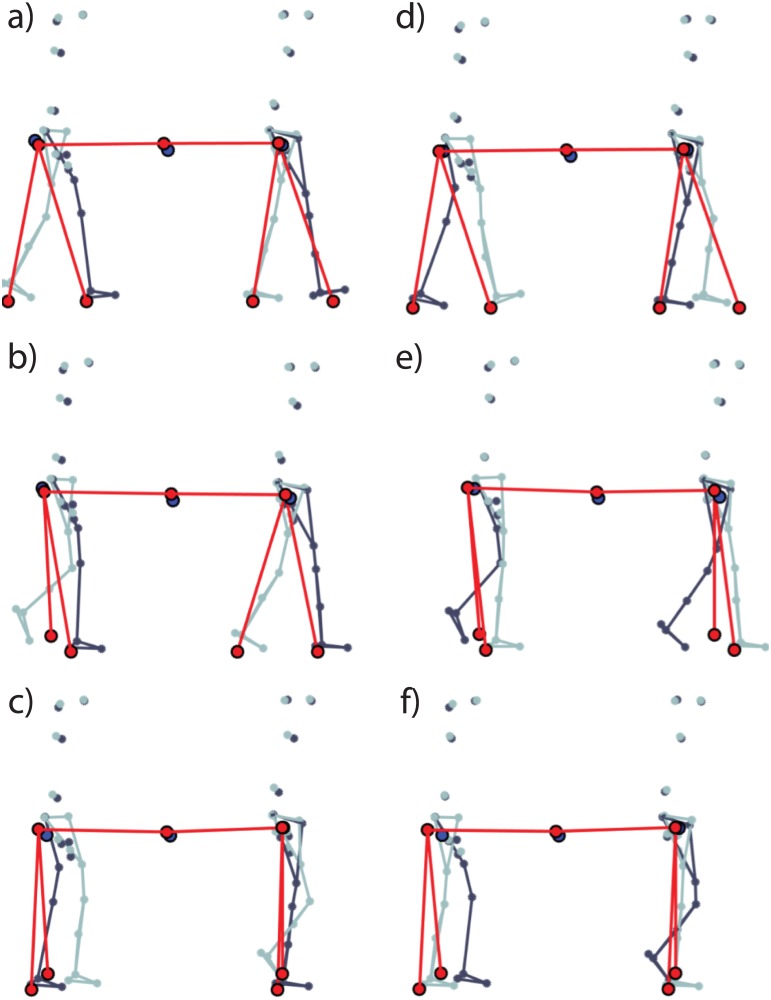
Snapshots of the experiments versus the simulations (Trial 4, Pair 02). From the top to the bottom, the first and second columns represent the temporal sequence of movements of a paired trial (from frame ‘a’ to frame ‘f’). The pictures show the results both from the experimental data and model data obtained by implementing the ‘paired trial fitting’ (see Subsection 5.1). Red dots refer to the modeled CoMs, the pivot points and the carried mass, and they are all connected with single red lines. Blue dots represent the CoMs of each subject and that of the table. Finally, grey dots represent subjects’ markers recorded during the experiments (dark grey is used to show the right side of the subject while light grey indicates the left side). Only the lower limb grey dots have been connected to better represents subjects’ legs and feet.

As an example, the foot-fall patterns in the experiments and the simulations are presented in Fig 11 for Pair 02 (similar results are obtained for other pairs), where we can observe ‘Trot’ like gait (on the left) and ‘Pace’ like gait (on the right). Since the model is 2-dimensional, we cannot distinguish between the trot and the pace gait. The distinction reported is based on the initial configuration of the two SLIPs: by assuming, for example, that the two pendulums start with the same leg (indicated as ‘right’), one can monitor such a leg with respect to the other (indicated as ‘left’). In this way if the two SLIPs reach a periodic gait while moving forward the same leg (‘right’ or ‘left’) at the same time, we can associate the resultant gait to a pace-like gait and otherwise to a trot-like gait. In Fig 11 the foot-fall pattern results of the paired trial fitting are presented. With the paired trial fitting we are able to reproduce trot and pace gaits observed in the experimental data with the same stepping frequency of the analyzed subject. To visualize how well the paired trial fitting can predict the experimental data, snapshots of the simulations versus the experimental data for Trial 4 of Pair 02 are presented in Fig 12 (see also S3 Video). With the solo trial fitting we are still able to reproduce the coordinated gaits (in particular, pace and trot gaits), but with a different stepping frequency compared to the one observed in the experimental data. Such a discrepancy is due to the model limitation in reproducing the reduction in forward velocity, as explained before for Fig 10.

## 6 Discussion

The presented work investigates the effects of a mechanical connection between two bipedal agents on their walking gaits, aiming to answer the following questions:

- Do humans alter their gaits when they are physically paired with another human through an object while one is walking in front of the other?
- Do humans temporally coordinate their movements when they are physically paired? If yes, do they exhibit typical “quadrupedal” gaits, and if so, which ones?
- To what extent the coordination is caused by the passive mechanical connection of the two bipedal system?
- Is it possible to replicate the experimental data with a paired Spring Loaded Inverted Pendulums (SLIPs) model?
- Is it possible to predict the behaviour of the paired walking after fitting each pendulum (SLIP) separately with data from the free (not paired) walking experiments?

To answer the above questions, we present human-human interactive locomotion experiments showing how the mechanical coupling causes changes in the human walking gait; in particular, we observe that such a mechanical coupling causes a statistically significant reduction in the preferred forward speed (*p* = 0.015), step length (*p* = 0.017), and a significant increase of the GCT (*p* = 0.02). This adaptation of the gait through reduction of speed and increase of GCT could be due to the fact that each subject tries to accomodate to the motion of the other subject which is detected by interaction forces, visual and acoustic information.

The vertical component of the CoM trajectory of each subject does not significantly change when paired (the worst dissimilarity measure value is *d* = 5.3 ⋅ 10^−6^). Moreover, we observe coordination in the foot-fall patterns of the paired subject in 72.5 ± 19.9% of each trial, on average. That is, on average in 72.5 ± 19.9% of each trial the subjects paired together through a mechanical link while walking for few steps have the same stride frequency and establish a constant phase lag between homologous legs. Indeed, the subjects reproduce some foot-fall patterns generally associated to quadrupedal type gaits (e.g. pace, trot, and diagonal-sequence). This inter-subject coordination can be considered as a starting point for further investigation through longer trials.

We also present a simplified model for the mechanical analysis of the interactive locomotion. The model can be used to infer some properties of the real system and to evaluate whether some human behaviours can be associated to passive mechanical properties. Performing the systematic search presented in Subsection 4.1, it is observed that the region of periodic behaviour as defined in Subsection 3.2 presented in terms of angles of attack and legs stiffness, enlarges once the two pendulums are paired compared to the case of solo walking (i.e., without any mechanical connections). This observation suggests that the mechanical link may be used by the bipedal agents as a way to make the system more robust.

Moreover, the simulation results show that the paired-SLIPs model can predict some characteristics of the gaits during the interactive locomotion. In particular, it is able to:

- reproduce the vertical CoM diplacement of human subjects: Experimental CoM vertical displacement has been reproduced by the model both with paired trial fitting optimization and with solo trial fitting optimization. The best and the worst fittings of the experimental CoM vertical displacement have been presented showing how well our simple model can reproduce the human behaviour in term of the CoM vertical displacement. Indeed, even in the worst fitting, the dissimilarity measure is very small (*d* = 1.8 ⋅ 10^−3^);
- predict the statistically negligible differance between subjects’ CoM vertical displacement during the solo and paired trials;
- predict and reproduce the emerging of some quadrupedal gait foot-fall patterns (in particular, pace and trot gaits);
- predict the preference for synchronized gaits in spite of different parameters of the agents;
- predict the different trends found in the experimental paired behaviours: if the two pendulums have the same initial velocity, they can either converge to another common velocity or they can keep the same walking speed. However, the latter case refers to identical modeled subjects (SLIPs) that can hardly reflect the reality. Finally, if two pendulums have different initial walking speeds, by choosing appropriate model parameters, they converge to another walking speed that is not the same as any of their initial velocities due to the energy dissipation.

However, the change in the forward velocity once the two subjects are paired, even for the ones with similar preferred speed during the solo trials, cannot be explained just by a mechanical effect with our simple model. As discussed before, according to the experiments, mechanical coupling the system has the effect of reducing the mean forward velocity for at least one subject within each pair. With the first algorithm, in which we optimize the system for the paired system parameters, we obtain a very good match between the experimental data and the simulation results even in terms of the forward velocity. However, when we do not optimize for experimental paired data, we cannot match the level of reduction in the forward velocity found in the experimental data for most of the subjects once paired with another one, and the forward velocity remains close to its value in the solo experimental trial, which was chosen to be the initial condition of the simulated paired system.

Moreover, the model does not demonstrate the D-S gait mainly because the two SLIP models must have the same motion due to dissipation.

These results suggest that some behavior of humans while carrying objects with other humans seem to be only due to the passive mechanical properties rather than high-level control strategies such as foot-placement. However, some other features of the emerging paired walking gait might be related to cognitive and/or psychological effects, that in the model may be represented by addition or subtraction of energy according to a specific control architecture. Probably, more complex motor control actions have to be considered in order to better understand this phenomenon.

## Supporting information

S1 Appendix Real conditions score and synchronization score description. (TEX)

S1 Video Paired-SLIPs model with periodic walking gait. The video shows a simulation of the paired-SLIPs model where the right choice of parameters allow the system to converge a periodic walking gait.
(MP4)

S2 Video Paired-SLIPs model with unstable walking gait. The video shows a simulation of the paired-SLIPs model where the wrong choice of parameters does not allow the system to converge a periodic walking gait.
(MP4)

S3 Video Comparison between paired-SLIPs model and experimental data. The video shows the results both from the experimental data and model data obtained by implementing the ‘paired trial fitting’ (see Subsection 5.1). Red dots refer to the modeled CoMs, the pivot points and the carried mass, which are all connected with single red lines. Blue dots represent the CoMs of each subject and that of the table. Finally, grey dots represent the markers placed on the subjects recorded during the experiments (dark grey is used to show the right side of the subject while light grey indicates the left side). In this case only the lower limb grey dots have been connected to better represent the subjects’ legs and feet.
(MP4)

S1 Fig Percentage of gait types. Relationship among the percentages of the different types of gait (pace gait in light blue, trot gait in blue and diagonal-sequence in grey) and three walking gait parameters: forward velocity (on the top), gait cycle time (in the middle) and step length (on the bottom). Such a relationship is evaluated for each pair (from Pair01, indicated with one star, to Pair07, indicated with seven stars).
(EPS)

S2 Fig Vertical and forward interaction forces. In this figure an evaluation of the vertical and forward interaction forces for Pair01 is shown. The four plots refer to four force sensors (Optoforce, OMD-30-FE-450N) placed in each handle of the strecher-like object that was used for the experiments. Sensor 1 and 3 were placed at the Subject A side (Sensor 1 on the right and Sensor 3 on the left), while Sensor 2 and 4 were placed at the Subject B side (Sensor 2 on the right and Sensor 4 on the left).
(EPS)

S1 Ethic Statement Ethic statement approved by the EPFL human research ethics committee (HREC No 004-2015). (PDF)
