# Supplementary material for: Interactive locomotion: Investigation and modeling of physically-paired humans while walking
Source: PLoS One. 2017 Sep 6;12(9):e0179989. doi: 10.1371/journal.pone.0179989 (PMC5587243; doi:10.1371/journal.pone.0179989)
Supplement: S1 Ethic Statement — (PDF) [file pone.0179989.s007.pdf]

ÉCOLE POLYTECHNIQUE
FÉDÉRALE DE LAUSANNE

Research Office
EPFL VPAA-DAR, Station 7, CH-1015 Lausanne
Website: http://research-office.epfl.ch/
Phone : +41 (0) 21 693 49 77
Fax : +41 (0) 21 693 55 83
E-mail : research.office@epfl.ch

EPFL HUMAN RESEARCH ETHICS COMMITTEE

Request for opinion on ethical acceptability of projects undertaken by researchers at EPFL

DECISION

HREC No: : 004-2015/21.07.2015
Office use only

Applicants' Name: Auke IJspeert and Jessica Lanini

Title of the project: CogIMon

For EPFL Research Ethics Committee use only:

Review date: 11.08.2015

Outcome: ☒ Approval (with comments)

☐ Approval Declined

Applicant informed (date): 26.08.2015

Prof. Andreas Mortensen

Chair Human Research Ethics Committee EPFL

Research Office
EPFL VPAA-DAR, Station 7, CH-1015 Lausanne
Website: http://research-office.epfl.ch/
E-mail : research.office@epfl.ch

Recommendations and comments from the reviewers

Reviewer 1

I contacted co-applicant Jessica Lanini to clarify a few details. She gave me the following clarifications:

- §B6: "personal data" (i.e. concerning participant's private life) will not in fact be collected. Data collected from participants will be physical measures (e.g. weight, height) or general information (gender, age, etc.), which will be irreversibly anonymized. Therefore, the protocol does not involve any relevant risk to participant's privacy.
- §C4: after initial contact by email, individuals willing to participate in the study will be invited to the BioRob Lab to perform the experiment. Before the experiment, the researcher will make sure that the person has fully understood the aim of the research, as well as other relevant information detailed in the information sheet. The participant will have the opportunity to ask for clarifications, and then may sign the consent form.

I suggested a couple of amendments to the information sheet and to the email that will be sent out for recruitment. Co-applicant Lanini agreed to make these corrections in the final version of these documents:

1) Information sheet:

   Linking information about risks (i.e. "*What are the risks*") to insurance of EPFL for any possible damage will increase the coherence of the information sheet and facilitate the participant's understanding.

2) Recruitment email:

   The email is addressed to students only. However, according to selection criteria specified in the protocol, participants might be from 18 to 40 years old. In order to meet selection criteria, the email should state that researchers are looking for: "students or EPFL collaborators".
   Moreover, the email should specify that the payment (20.- chf/h) is awarded to participants as compensation for the time spent in the research. I suggest to avoid expressions such as "votre participation, récompensée par 20 chf/h", or "you will help us to learn about... and you will receive 20 chf/h", since they seem to suggest that the money is given as a "salary" or as an incentive for participating.
   An alternative way to encourage participation may be to explain in the email that the research aims to contribute to the development of robotic assistants.

ÉCOLE POLYTECHNIQUE
FÉDÉRALE DE LAUSANNE

Research Office
EPFL VPAA-DAR, Station 7, CH-1015 Lausanne
Website: http://research-office.epfl.ch/
E-mail : research.office@epfl.ch

Reviewer 2

B6: I would suggest giving more details concerning the way data will be anonymized

C5: I would suggest giving more details concerning the assessment of healthy state of the research subjects that should/could? be based on a medical certificate. Furthermore, indicate where medical assistance could take place in case health problem happen?

C6: I would suggest justifying the amount of CHF 20 per hour (what kind of compensation?)

Reviewer 3 (legal)

No comments – approval

Reviewer 4

No comments - approval

Reviewer 5

The project is OK for a approval but
• there is too little mention of how data to be anonymized and how are data to be stored and made openly available (DMP)?
• the phrasing ("receive" or "récompense") concerning the 20 CHF and the fact that it is paid on an hourly basis suggests that it is a salary; I would change the wording if this is not so.

Research Office
EPFL VPAA-DAR, Station 7, CH-1015 Lausanne
E-mail : research.office@epfl.ch

SECTION A APPLICATION DETAILS

A1	**Project : CogIMon**	
	Date of Submission: 15.07.2015	Proposed Starting Date: 01.09.2015
		Proposed End Date: 30.09.2015

A2	**Principal Investigator**<br>Please attach the curriculum vitae and publication list (of the past 3 years).	
	Full Name: Jessica Lanini<br>(Supervisor Prof. Auke Ijspeert)	Position Held: PhD student
	Address: EPFL STI BIOROB, INN 239,<br>Station 14, CH-1015 Lausanne, Switzerland	Email: jessica.lanini@epfl.ch<br>Phone: 021 693 26 58

A3	**Involved collaborators**<br>Please attach the curriculum vitae of all collaborators.	
	Full Name: Mostafa Ajallooeian	Position Held: Post-doc
	Address: EPFL STI BIOROB, INN 235,<br>Station 14, CH-1015 Lausanne	Email: mostafa.ajallooeian@epfl.ch<br>Phone: 021 693 26 76
	Full Name: Cole Simpson	Position Held: Academic Guest
	Address: EPFL STI BIOROB, INN 212,<br>Station 14, CH-1015 Lausanne, Switzerland	Email: colesimpson90@gmail.com<br>Phone: 078 832 80 18
	Full Name:	Position Held:
	Address:	Email:<br>Phone:

A4	**Funding Sources:**					
	Funding applied for:	Yes ☐	No ☐	Not appli able ☐		
	Funding secured:	Yes ☑	No ☐	Not applicable ☐		

Name of organization from which funding has been obtained/sought?
EU-funded research project in the Horizon 2020 Work Programme.

Signature of the Principal Investigator: [signature] Date:

Co-Signed by Supervisor where PI is a Student: [signature] Date:

Research Office
EPFL VPAA-DAR, Station 7, CH-1015 Lausanne
E-mail : research.office@epfl.ch

SECTION B DETAILS OF THE PROJECT ACTIVITIES

B1 **Project Summary**

Please provide a brief summary of the project in lay terms outlining the intended value of the project, giving necessary scientific background (max 500 words).

In everyday life, it is sometimes necessary to move large or heavy objects that require the assistance of several human operators. However, human assistants are not always available or do not have sufficient capability to accomplish the task (eg. a task requiring superhuman strength). In these situations, it would be incredibly convenient to have a robotic assistant that is always available and is capable of working with humans or other robots to accomplish a wide variety of related tasks.

Many problems emerge when two agents are physically coupled together including stability and control (ie. how do agents communicate and direct co-workers towards task accomplishment). However, humans are able to gracefully and robustly accomplish similar coupled locomotion tasks.

We propose to perform a series of experiments that will allow us to examine how humans work cooperatively to accomplish a physically coupled locomotion task in order to understand how to best design assistive robot controllers. In particular, we wish to examine:

1) Do humans alter their gait patterns when they are physically coupled with another human?
2) How are interaction forces used to communicate task goals?
3) Do interaction forces contribute to stability, robustness, or task performance?

In literature surveys and prior experiments, our collaborators and we have found that humans tend to temporally coordinate their movements with each other. This has inspired an additional set of research questions that we would like to investigate including:

4) Do humans temporally coordinate their movements when they are physically coupled?
5) Does temporal coordination serve any mechanical purpose (eg. minimize interaction forces, facilitate stability or robustness)?

The experiments' results will be used in order to discern stereotypical patterns of human gait during coupled locomotion tasks. We will then attempt to reproduce these patterns in computer simulation using state-of-the-art models of human locomotion and motor control in order to understand the underlying neurological and control principles. We will then design new controllers for humanoid robots that will allow them to safely, stably, and robustly accomplish coupled locomotion tasks.

B2 **Research Protocol**

Characterize your research protocol, type of procedure and/or research methodology (e.g. observational, survey research, experimental). Give details of any samples or measurements to be taken (max 500 words)

Research Office
EPFL VPAA-DAR, Station 7, CH-1015 Lausanne
E-mail : research.office@epfl.ch

Attach any questionnaires, psychological tests, etc.

This experiment will test groups of 3 subjects at a time which each group's total time commitment being approximately 90 minutes. In order to achieve statistically significant results, we anticipate that 15 subjects will be required.
The necessary equipment is made up of:

- a stretcher-like object that subjects will carry;
- four 3D force sensors in order to detect forces of interaction;
- one inertia measurement units (IMU) used to evaluate the orientation changes of the stretcher-like object;
- two markers placed on one foot of each subject;
- one camera;
- two headphones.

Each group will be split into three couples (subjects 1 and 2, 2 and 3, and 1 and 3). Each couple will be asked to perform 3 trials:

Trial 1 (gait analysis baseline): One subject is placed behind a starting point. One of the experimentors will ask the subject to start walking. The subject will walk freely for 200 m on a flat terrain while their movements are recorded with a video camera. The subject will be notified when to stop at the end of the trial.

Goal of the trial:
Measure baseline gait parameters (GCT, frequency, stride length).

Trial 2 (coupled task performance): Subjects are placed behind a starting point, one behind the other while carrying a stretcher-like object. One of the experimentors will ask the subjects to start walking. Subjects will carry the stretcher (<5 kg total) for 200 m on a flat terrain where an experimentor will tell subjects to stop. The trial will be repeated with the reverse subject order (the subject that was in front at first will be in the rear).

Goal of the trial:

- We will evaluate the gait parameters and compare them with those from Trial 1 (question 1-2). In doing this comparison, we can directly examine how characterists of normal walking (a well-studied behavior) are modified when subjects perform this task.
- We will search for correlations between interaction forces and walking behavior (eg. walking speed, stepping frequency, and stride length) (question 2).
- We will analyze whether any characteristics of the two subjects temporally coordinate when they are physically coupled by the stretcher (question 3).
- We will examine whether initial conditions (eg. which foot the subject moves first or the timing of the first step) effect temporal coordination between the two subjects (question 4).
- We will search for relationships between measured (eg. interaction forces or step timing) or imposed parameters (eg. position relative to the other subject) and apparent dominance/leadership in the task (the subject whose

preferred gait parameters are dominant in determining the coupled gait characteristics; eg. the subject whose preferred walking speed is closer to the walking speed of the two subjects carrying the stretcher).

Trial 3(physical connection + acoustic feedback):
Subjects are placed behind a starting point, one behind the other one while carrying the stretcher-like object. Each subject will be given a pair of headphones and will be provided a tempo via a metronome perfectly distinguishable by any test subject. The subjects will be asked to match their footfalls with the metronome beats provided. The two subjects will be given beats that have some time delay between them and they will be asked to walk 200 m. This trial will be repeated for 3 different time delays between the beats provided to each subject.

Goal of the trial:
We will be able to evaluate the mechanical advantages or disadvantages of temporally coordinating movements with a co-worker in a coupled locomotion task.

Trial 4 (physical connection + acoustic feedback):
Subjects are placed behind a starting point, one behind the other one while carrying a stretcher-like object on a flat terrain. Each subject will be given a pair of headphones and will be provided a tempo via a metronome. The subjects will be asked to match their footfalls with the metronome beats provided. The two subjects will be given beats that have different frequencies (less than one order of magnitude) and they will be asked to walk 200m.

Goal of the trial:
We will analyse the effect of different stepping frequencies on measured task parameters (eg. interaction forces and table orientation) that are correlated to possible optimal task performance measures (eg. minimize energy expenditure of the system, maximize robustness of the system).

Trial 5 (Effect of haptic communication on temporal coordination):
Subjects are place behind a starting point, one behind the other one while carrying a stretcher-like object on a flat terrain. One of the subjects (randomly selected) will be blindfolded and given sound-isolating headphones in order to control for visual and auditory feedback. Subjects will then be asked to walk 200m. The blindfolded subject will be guided by the physical connection with the other subject and will be closely monitored by the experimenters to ensure safety.

Goal of the trial:
The goal of this trial is to determine the importance of interaction forces in communicating task-level goals and information necessary to temporally coordinate movement patterns.

Research Office
EPFL VPAA-DAR, Station 7, CH-1015 Lausanne
E-mail : research.office@epfl.ch

Questionnaire

1. Did you consciously try to synchronize your steps with your partner during the experiment?
2. Did you feel as though the shared object restricted your movement?
3. Did you try to maintain a constant orientation of the stretcher?
4. Did you consciously try to move the stretcher during the experiment?
5. Do you have any suggestions for ways to improve this experiment?

Research Office
EPFL VPAA-DAR, Station 7, CH-1015 Lausanne
E-mail : research.office@epfl.ch

**B3**	**Where will the study take place (name of institution/department)?**<br>Is the study compliant with the CH Federal law on data protection ("*Loi fédérale sur la protection des données*" – RS 235.1)? If the study is to be carried out in another country, what steps have been taken to secure research and ethical permission in the study country?
	The study will take place at Centre Sportif Universitaire de Dorigny (CSUD), Lausanne.

**B4**	**Have collaborating institution/departments whose resources will be needed been informed and agreed to participate?**<br>*Attach all relevant documentation.*
	Not applicable

B5 **How and to whom the results will be disseminated, including communication of results with research participants?**

Published in peer reviewed journals, or as an Open Access report	☑
Presented at conferences	☑
Professional or anizations, peer research rs, olicy makers	☐
Brochure, flyer to participants, interested parties	☐
Internet	☐
Other	☐

	Yes	No
Are there any restrictions by e.g. data providers on the dissemination of the research findings?	☐	☑

If yes, please describe the nature of the restrictions.
Cliquez ici pour taper du texte.

**B6**	**Outline any ethical issues that might be expected from the proposed research and how they will be addressed.**<br>You might want to refer to issues such as inform consent, the degree of confidentiality and anonymity that can be assured, anonymization procedures, protection from harm, right to withdraw, the storage of sensitive data etc.
	Our research will involve personal data collection and further processing of them. For this reason each subject will be asked to sign an inform consent and the anonymity of the procedure will be assured.

Research Office
EPFL VPAA-DAR, Station 7, CH-1015 Lausanne
E-mail : research.office@epfl.ch

SECTION C DETAILS OF PARTICIPANTS

C1 Participants to be studied

Number of volunteers:	15
Upper age limit	40 years old
Lower age limit	18 years old

Please justify the age range and sample size:
Our study requires the use of healthy adults (>18). Because the study involves some physical activity (walking outdoors carrying a light load), we would like to exclude subjects over the age of 40. We anticipate that approximately 15 subjects will be necessary to achieve statistically significant results.

Will the participants participate on a fully voluntary basis? Yes: ☑ No: ☐

Will EPFL students be involved as partici ants in the study? Yes: ☑ No: ☐

Please explain how you will bring to the attention of the participants their right to withdraw themselves, their samples and data from the study without penalty?

Participants' rights will be enumerated on the information sheet when they are first contacted regarding their participation in the study. Their rights will be repeated prior to the beginning of the experiment.

C2 If data or information held by a third party will be used, please explain how you will obtain these data.

You should confirm that the information will be obtained in concordance with the CH Federal law on data protection ("Loi fédérale sur la protection des données" – RS 235.1).

The information will be obtained in concordance with the CH Federal law on data protection ("Loi fédérale sur la protection des données" – RS)

C3 Will the research include children or vulnerable adults?

Such as people with learning difficulties; individuals with mental health problems; elderly, prisoners, other vulnerable groups e.g. dementia, psychological disorders etc., please specify and justify

Yes: ☐ No: ☑

If yes, please specify how you will ensure that participants in these groups are competent to give consent to take part in this study.
Cliquez ici pour taper du texte.

C 4	**How will the participants in the study be**
	(i) Selected?<br>Subjects will be selected according to their availability and satisfaction of the inclusion criteria.<br><br>(ii) Recruited? *Attach all relevant documentation.*<br>They will be recruited by email:

English below

Appel à participation Au laboratoire de Biorobotique (Biorob, EPFL) nous tentons d'évaluer les interactions entre humains lors de tâches collaboratives couplées (par exemple, lors du transport d'une table par deux personnes) et nous sommes à la recherche de volontaires pour une étude utilisateur portant sur cet aspect. Durant cette étude, les participants devront déplacer, en marchant, un objet extensible par groupe de deux et, par la suite, remplir un questionnaire.
La durée globale de l'étude ne devrait pas dépasser **90 minutes**. Nous recherchons des **étudiants toutes filières confondues**. Aucune compétence particulière n'est requise. L'étude se déroulera dans les locaux du **BioRob** pendant la semaine du **1 au 30 Septembre 2015**. Votre participation, récompensée par **20 CHF/h**, nous aidera à améliorer notre système. Si vous êtes intéressés, merci de répondre à cet email et nous vous recontacterons prochainement.

Sincères salutations,

Jessica Lanini

****

Call for participants At the Biorobotics Laboratory (BioRob, EPFL) we are evaluating human-human interaction in shared locomotor tasks and are looking for participants to take part in a our study. The study will focus on aspects of interaction when participants walk together while carrying a stretcher-like object. Subjects will also be asked to fill out a short questionnaire afterwards. Overall, the experiment will take about **90 minutes**. We are looking for **students of all backgrounds**. There are no special skills required. The study will take place at BioRob, EPFL between **1 and 30 September 2015**. Through you participation, you will help us to learn about human-human interaction and locomotion and you will receive **20 CHF/hr**. If you are interested, please reply to this email and we will get in touch with you soon.

Thank you very much,

Jessica Lanini.

Jessica Lanini
Research Assistant - PhD student

EPFL - STI - IBI - BIOROB
INN 239 (bâtiment INN)
Station 14
CH-1015 Lausanne

Phone: +41 78 723 15 52
e-Mail: jessica.lanini@epfl.ch
Web: http://biorob.epfl.ch/

(iii) Informed? How much time will the participants have to make a decision

	between the complete information and the beginning of the study?
	Subjects will be given at least a week to decide to withdraw from the study after initial contact. They will be reminded of their rights orally immediately before the experiment.

**C5**	**What criteria will be used for inclusion and exclusion of participants?**
	(i) Inclusion criteria
	Willing and available healthy adults between the ages of 18 and 40 with no known neuromotor disfunctions will be included in this study.
	(ii) Exclusion criteria
	Subjects with known health or neuromotor problems will not be included in this study.

**C6**	**Will payment or any other incentive, such as gifts, be made to the participants?**
	Yes: ☑ No: ☐
	If yes, please give details (level of payment, source of payment, source of gifts).<br>Subjects will be compensated at a rate of 20 chf per hour.

Research Office
EPFL VPAA-DAR, Station 7, CH-1015 Lausanne
E-mail : research.office@epfl.ch

SECTION D INFORMATION SHEET(S) FOR PARTICIPANTS

**D 1**	**Please attach a copy of the written participant Information sheet.**<br>If no Information sheet is to be given to the participants, please justify.

Research Office
EPFL VPAA-DAR, Station 7, CH-1015 Lausanne
E-mail : research.office@epfl.ch

INFORMATION SHEET FOR PARTICIPANTS IN RESEARCH STUDIES

You will be given a copy of this information sheet.

Project Title: CogIMon
This study has been approved by the EPFL Research Ethic Commitee (HREC No: ___)

Name:
Work Address:
Contact Details:

We would like to invite _______________ to participate in this research project.

Details of the study:
The study is focused on the evaluation of the behavior that characterizes human-human collaboration while carrying an object so, when they are physically coupled.
So you walk while carry a stretcher-like object with another participant from one initial position to a final one and then come back.
We are interested in the evaluation of the foot-fall pattern adopted by each participant during the task, the orientation of the object and the forces of interaction.
At the end of the trial you will be asked to answer to a simple questionnaire.
What are the risks?
There are no anticipate risks associated with participing in this study.

Please ask us if there is anything that is not clear or if you would like to receive more information.
It is up to you decide whether you will take part or not; choosing not to take part will not disadvantage you in any way. As a participant you have the right to withdraw from the study at any time without giving a reason or facing negative consequences.
All data will be collected and stored safely and reported in an anonymous form, in accordance with the CH Federal law on data protection ("Loi fédérale sur la protection des données" – RS 235.1). Only the principal investigator and/or the members of the Research Ethics Committee have access to the original data under strict confidentiality.
Possible damage to our health, which is directly related to the above study and is demostrably the fault of EPFL, is covered by the general liability insurance of EPFL (insurance policy no. 30/5.006.824 of the Baloise Insurance). However, beyond the before mentioned, the health insurance and the accident insurance is in the responsibility of the participant.

D2	**How to prepare Information sheet(s) for participants?**
	(i) In general:<br>a. Information sheet(s) should be written in a simple, understandable language. For children a separate information sheet should be written in an understandable language adapted to their age.<br>b. Participants should be directly addressed. E.g. "You will be informed during the whole project…" and not "The participant will be informed…".<br>(ii) The Information sheet(s) should contain the following items:<br>a. Aims of the research and possible benefits<br>b. Risks for the participants (in a separate paragraph)<br>c. Research procedure/methods formulated in an understandable way<br>d. Source of funding of the research<br>e. Who you are recruiting; indications of selection and exclusion criteria<br>f. What will happen if they participate (when, where, how long etc.)<br>g. Advantages and disadvantages for participants/risks<br>h. Sort and extent of a possible compensation. In the case of no compensation, this should be explicitly stated.<br>i. Right of withdrawal<br>j. Arrangements of ensuring anonymity and confidentiality, data access-processing-storage-deletion<br>k. Insurance coverage<br>l. Contact information

Research Office
EPFL VPAA-DAR, Station 7, CH-1015 Lausanne
E-mail : research.office@epfl.ch

Example in English:

INFORMATION SHEET FOR PARTICIPANTS IN RESEARCH STUDIES

You will be given a copy of this information sheet.

Project Title:Cliquez ici pour taper du texte.

This study has been approved by the EPFL Research Ethics Committee (HREC No: Cliquez ici pour taper du texte.)

Name: Cliquez ici pour taper du texte.

Work Address: Cliquez ici pour taper du texte.

Contact Details: Cliquez ici pour taper du texte.

We would like to invite Cliquez ici pour taper du texte. to participate in this research project.

Details of the study:

Cliquez ici pour taper du texte.

What are the risks?

Cliquez ici pour taper du texte.

Please ask us if there is anything that is not clear or if you would like to receive more information.

It is up to you to decide whether you will take part or not; choosing not to take part will not disadvantage you in any way. As a participant you have the right to withdraw from the study at any time without giving a reason or facing negative consequences.

All data will be collected and stored safely and reported in an anonymous form, in accordance with the CH Federal law on data protection ("Loi fédérale sur la protection des données" – RS 235.1). Only the principal investigator and/or the members of the Research Ethics Committee have access to the original data under strict confidentiality.

Possible damage to our health, which is directly related to the above study and is demonstrably the fault of EPFL, is covered by the general liability insurance of EPFL (insurance policy no. 30/5.006.824 of the Baloise Insurance). However, beyond the before mentioned, the health insurance and the accident insurance is in the responsibility of the participant.

Research Office
EPFL VPAA-DAR, Station 7, CH-1015 Lausanne
E-mail : research.office@epfl.ch

Example in French:

NOTICE D'INFORMATION AUX PARTICIPANTS À UN PROJET DE RECHERCHE

Un exemplaire de cette notice d'information doit vous être remis.

Nom du projet : Cliquez ici pour taper du texte.

Ce projet de recherche a été approuvé par le Comité d'éthique de la recherche humaine de l'EPFL (HREC No: Cliquez ici pour taper du texte.).

Nom : Cliquez ici pour taper du texte.

Adresse professionnelle : Cliquez ici pour taper du texte.

Comment nous contacter : Cliquez ici pour taper du texte.

Nous aimerions inviter Cliquez ici pour taper du texte. à participer à ce projet de recherche.

Détails du projet :

Cliquez ici pour taper du texte.

Quels sont les risques ?

Cliquez ici pour taper du texte.

Vous êtes invité à nous demander toute clarification nécessaire ou toute information complémentaire.

La décision de participer ou non au projet vous revient. Le fait de ne pas participer ne vous désavantagera pas. En tant que participant, vous avez le droit de vous retirer du projet à tout moment, sans aucune conséquence négative pour vous.

Toutes les données traitées dans le cadre du projet de recherche seront collectées et sauvegardées de manière sécurisée et anonyme, conformément à la Loi fédérale sur la protection des données (RS 235.1). Seuls le chercheur principal et/ou les membres du Comité d'éthique de la recherche humaine de l'EPFL auront accès aux données originales, et seront soumis à une obligation de stricte confidentialité.

Un éventuel dommage à votre santé, directement causé par le projet de recherche ci-dessus et découlant manifestement d'une faute de l'EPFL, est couvert par l'assurance responsabilité civile de l'EPFL (police d'assurance N° 30/5.006.824 de Bâloise Assurances). Cependant, dans tout

Research Office
EPFL VPAA-DAR, Station 7, CH-1015 Lausanne
E-mail : research.office@epfl.ch

autre cas, il est de votre responsabilité de conclure une assurance maladie et accident.

Research Office
EPFL VPAA-DAR, Station 7, CH-1015 Lausanne
E-mail : research.office@epfl.ch

SECTION E	CONSENT FORM(S)

**E 1**	**Is written consent for participation in the study to be obtained?**<br><br>Yes: ☑ No: ☐<br><br>If yes, please attach a copy of the consent form to be used. *Consent form examples see below.*<br><br>If no, please explain why

INFORMED CONSENT FORM FOR PARTICIPANTS IN RESERACH STUDIES

Please complete this form after you have read the Information Sheet and received an explanation about the research.

Project Title: CogIMon
Principal Investigator: Jessica Lanini
Participant:

Thank you for your interest in taking part in this research. Before you agree to take part, the person organising the research must explain the project to you. If you have any questions arising from the Information Sheet or explanation already given to you, please ask the resercher before you decide to join in. You will be given a copy of this Consent Form to keep and refer to any time.

Participant's Stantement

I

- agree the research project named above has been explained to me to my satisfaction and I agree to participate in this study on a voluntary basis.

- understand that if I decide at any time that I no longer wish to take part in this project I can notify the researchers involved and withdraw from the study without giving reasons and without any negative consequences.

- have read the Information Sheet for participants and I have received a copy of the Information Sheet and Consent Form.

- have been informed that all data will be collected and stored safely and reported in an anonymous form, in accordance with the CH Federal law on data protection ("Loi fédérale sur la protection des données" – RS 235.1).

- agree that the principal investigator and/or the members of the Research Ethics Committee have access to the original data under strict confidentiality.

- have been informed that possible damage to my health, which is directly related to the above study and is demonstrably the fault of EPFL, is covered by the general liability insurance of EPFL (insurance policy no. 30/5.006.824 of the Baloise Insurance). However, beyond the before mentioned, my health insurance and/or accident insurance will apply.

The extra copy of this consent form is for you to keep.

Date ***Signature***

Research Office
EPFL VPAA-DAR, Station 7, CH-1015 Lausanne
E-mail : research.office@epfl.ch

**E2**	**What special arrangements have been made to deal with the issues of consent and assent for vulnerable participants?** Such as people with learning difficulties; individuals with mental health problems; elderly, prisoners, other vulnerable groups e.g. dementia, psychological disorders etc.
	E.g. is parental or guardian agreement to be obtained and if so in what form?<br><br>In case of vulnerable participants a written parental agreement would be necessary in order to deal with the issue of consent.

**E3**	**How to prepare Consent Form(s) for participants?**
	Statements which *might* be included if appropriate (non-exhaustive list):<br>• I understand that my participation will be taped/video recorded and I consent the use of this material as part of the project.<br>• I agree to be contacted in the future by EPFL researchers who would like to invite me to participate in follow-up studies.<br>• I understand that the information I have submitted will be published as a report and I will be sent a copy. Confidentiality and anonymity will be maintained and it will not be possible to identify me from any publications.

Research Office
EPFL VPAA-DAR, Station 7, CH-1015 Lausanne
E-mail : research.office@epfl.ch

Example in English:

INFORMED CONSENT FORM FOR PARTICIPANTS IN RESEARCH STUDIES

Please complete this form after you have read the Information Sheet and received an explanation about the research.

Project Title: Cliquez ici pour taper du texte.

Principal Investigator (Name and First Name): Cliquez ici pour taper du texte.

Participant (Name and First Name): Cliquez ici pour taper du texte.

Thank you for your interest in taking part in this research. Before you agree to take part, the person organising the research must explain the project to you.

If you have any questions arising from the Information Sheet or explanation already given to you, please ask the researcher before you decide to join in. You will be given a copy of this Consent Form to keep and refer to at any time.

Participant's Statement

I

- agree the research project named above has been explained to me to my satisfaction and I agree to participate in this study on a voluntary basis.
- understand that if I decide at any time that I no longer wish to take part in this project I can notify the researchers involved and withdraw from the study without giving reasons and without any negative consequences.
- have read the Information Sheet for participants and I have received a copy of the Information Sheet and Consent Form.
- have been informed that all data will be collected and stored safely and reported in an anonymous form, in accordance with the CH Federal law on data protection ("Loi fédérale sur la protection des données" – RS 235.1).
- agree that the principal investigator and/or the members of the Research Ethics Committee have access to the original data under strict confidentiality.
- have been informed that possible damage to my health, which is directly related to the above study and is demonstrably the fault of EPFL, is covered by the general liability insurance of EPFL (insurance policy no. 30/5.006.824 of the Baloise Insurance). However, beyond the before mentioned, my health insurance and/or accident insurance

Research Office
EPFL VPAA-DAR, Station 7, CH-1015 Lausanne
E-mail : research.office@epfl.ch

will apply.

The extra copy of this consent form is for you to keep.

Signature:	
Date:	

Research Office
EPFL VPAA-DAR, Station 7, CH-1015 Lausanne
E-mail : research.office@epfl.ch

Example in French:

FORMULAIRE DE CONSENTEMENT ÉCLAIRÉ POUR PARTICIPANT À UN PROJET DE RECHERCHE

Merci de remplir ce formulaire après avoir lu la notice d'information aux participants et avoir reçu des explications au sujet du projet de recherche

Nom du projet : Cliquez ici pour taper du texte.

Chercheur principal (nom et prénom): Cliquez ici pour taper du texte.

Participant (nom et prénom): Cliquez ici pour taper du texte.

Nous vous remercions pour l'intérêt que vous portez à ce projet de recherche. Avant que vous acceptiez de participer, la personne responsable du projet de recherche doit vous expliquer en quoi il consiste.
Si vous avez des questions au sujet de la notice d'information aux participants ou des explications qui vous ont été données, merci de les poser aux chercheurs avant de décider de participer. Une copie de ce formulaire de consentement éclairé doit vous être donnée afin que vous puissiez la garder et la consulter en tout temps.

Déclarations du participant

Je certifie

- Que le projet de recherche ci-dessus m'a été expliqué à mon entière satisfaction, et que j'accepte d'y participer de manière volontaire.
- Comprendre que je peux décider à tout moment de ne plus participer au projet de recherche sans donner de raisons et sans aucune conséquence négative pour moi. Dans ce cas, il suffit de communiquer ma décision aux chercheurs.
- Avoir lu la notice d'information aux participants et avoir reçu une copie de la notice d'information aux participants et du formulaire de consentement éclairé.
- Avoir été informé(e) que toutes les données traitées dans le cadre du projet de recherche seront collectées et sauvegardées de manière sécurisée et anonyme, conformément à la Loi fédérale sur la protection des données (RS 235.1).
- Accepter que le chercheur principal et/ou les membres du Comité d'éthique de la recherche humaine de l'EPFL auront accès aux données originales, et seront soumis à une obligation de stricte confidentialité.
- Avoir été informé(e) qu'un éventuel dommage à ma santé, directement causé par le

Research Office
EPFL VPAA-DAR, Station 7, CH-1015 Lausanne
E-mail : research.office@epfl.ch

projet de recherche ci-dessus et découlant manifestement d'une faute de l'EPFL, est couvert par l'assurance responsabilité civile de l'EPFL (police d'assurance N° 30/5.006.824 de Bâloise Assurances). Cependant, dans tout autre cas, il est de ma responsabilité de conclure une assurance maladie et accident.

Une copie de ce formulaire est pour vous.

Signature:	
Date:	

Research Office
EPFL VPAA-DAR, Station 7, CH-1015 Lausanne
E-mail : research.office@epfl.ch

SECTION F CHECKLIST

**Documents to be Attached to the Application Form** (if applicable)	Tick if attached	Tick if not relevant
Section A: Application Details		
• Curriculum vitae and publication list of PI	☐	☑
• Curriculum vitae of collaborators	☐	☑
Section B: Details of the Project		
• Questionnaire(s)/Psychological tests	☑	☐
• Relevant correspondence related to the involvement of collaborating institute(s)	☐	☑
Section C: Details on Participants		
• Recruitment documentation (e.g. advertisement)	☑	☐
Section D: Information Sheet(s) for participants		
• Participant(s) Information Sheet(s)	☑	☐
Section E: Consent Form(s)		
• Participant(s) Consent Form(s)	☑	☐
• Parental/guardian consent form for research involving participants under 18	☐	☑
